# Chinese herbal medicine for the treatment of Henoch–Schönlein purpura nephritis in children: A prospective cohort study protocol

**DOI:** 10.1097/MD.0000000000011064

**Published:** 2018-06-15

**Authors:** Jun Zhang, Jing Lv, Shuang Pang, Xiaohong Bai, Fang Yuan, Yubin Wu, Hong Jiang, Guanqi Yang, Shaoqing Zhang

**Affiliations:** a Affiliated Hospital of Liaoning University of Traditional Chinese Medicine; b Liaoning University of Traditional Chinese Medicine; c Department of Pediatric Nephrology, Shengjing Hospital of China Medical University; d First Affiliated Hospital of China Medical University, Shenyang, China.

**Keywords:** Chinese herbal medicine, cohort study, Henoch–Schönlein purpura nephritis, protocol

## Abstract

**Introduction:**

Henoch–Schönlein purpura nephritis (HSPN) involves the renal impairment of Henoch–Schönlein purpura and can easily relapse into life-threatening late nephropathy in severe cases. Although there is a lack of validated evidence for its effectiveness, Chinese herbal medicine (CHM) is one of the most commonly used methods in China to treat HSPN. It is thus need to report the protocol of a prospective cohort trial using CHM to investigate the effectiveness, safety and advantages for children with HSPN.

**Methods and analysis:**

This large, prospective, multicenter cohort study started in May 2015 in Shenyang. Six hundred children diagnosed with HSPN were recruited from 3 institutions and are followed-up every 2 to 4 weeks till May 2020. Detailed information of participants includes general information, history of treatment, physical examination, and symptoms of TCM is taken face-to-face at baseline.

**Ethics and dissemination:**

This study has received ethical approval from the ethics committee of institutional review board of the Affiliated Hospital of Liaoning University of Traditional Chinese Medicine (No.2016CS(KT)-002-01). Articles summarizing the primary results and ancillary analyses will be published in peer-reviewed journals.

**Trial registration:**

Clinical Trials Registration: NCT02878018.

## 1 Introduction

Henoch–Schönlein purpura (HSP) is a systemic disorder characterized by leukocytoclastic vasculitis involving the capillaries and deposition of IgA immune complexes.^[1]^ The ages of more than 90% of HSP patients are <10 years.^[2,3]^ Epidemiological studies have shown that Asians have a relatively higher incidence rate of HSP than Caucasians and Africans.^[2]^ Kidney injury, which is called Henoch–Schönlein purpura nephritis (HSPN), is one of the major manifestations and the primary cause of mortality from HSP.^[1]^ During the 4 to 6 weeks of initial disease presentation, approximately 40% of children with HSP progress to HSPN.^[4,5]^ Persistent purpura, severe abdominal symptoms, and an older age are 3 of the strongest risk factors for HSPN.^[6]^ Great efforts are needed to control the kidney injury for children with HSP who are at an increased risk of renal insufficiency.

However, therapies of Western medicine (WM) for HSPN, including corticosteroids, immunosuppressive treatment, and anticoagulant therapy, have the potential to cause adverse side effects and remain controversial.^[7–9]^ A clinical study demonstrated that early corticosteroid therapy does not prevent delayed nephritis in children with HSP.^[10]^ Immunosuppressive agents including cyclophosphamide have side effects such as oncogenesis, myelosuppression, hemorrhagic cystitis, and interstitial pneumonia.^[11,12]^ Moreover, the symptoms may recur easily after a long duration of treatment. Effective treatment for improving the long-term clinical management of HSPN remains an urgent need.

In China, the majority of HSPN patients resort to Chinese herbal medicine (CHM), acupuncture, and other traditional Chinese medicine (TCM) therapies.^[13,14]^ In TCM, HSPN is categorized as “purpura” or “hematuria.” In recent years, quite a few trials conducted in China have suggested that the use of CHM in conjunction with corticosteroids or immunosuppressive drugs generated additional positive effects,^[15–20]^ such as relieved blood hypercoagulable state, reduced proteinuria, and improved TCM signs and symptoms. These results indicated a potential role for CHM in the combined, consolidate, and maintenance treatment of HSPN. In this trial, we intend to assess the efficacy and advantage of the use of CHM for the treatment of children with HSPN.

## 2 Methods and analysis

### 2.1 Study design

This large, prospective, multicenter cohort study is being conducted in 3 institutions between May 2015 and May 2020. Six hundred participants are recruited and divided into 2 cohorts, with 300 individuals per cohort. All cohorts will undergo a 12-week treatment and a 12-month follow-up period. Participants will be asked to visit the clinicians every 2 weeks during the treatment period and once a month during the follow-up period. The feasibility and preciseness of the whole process are supervised by 1 to 2 supervisors.

### 2.2 Study settings and participants

**Figure 1:**
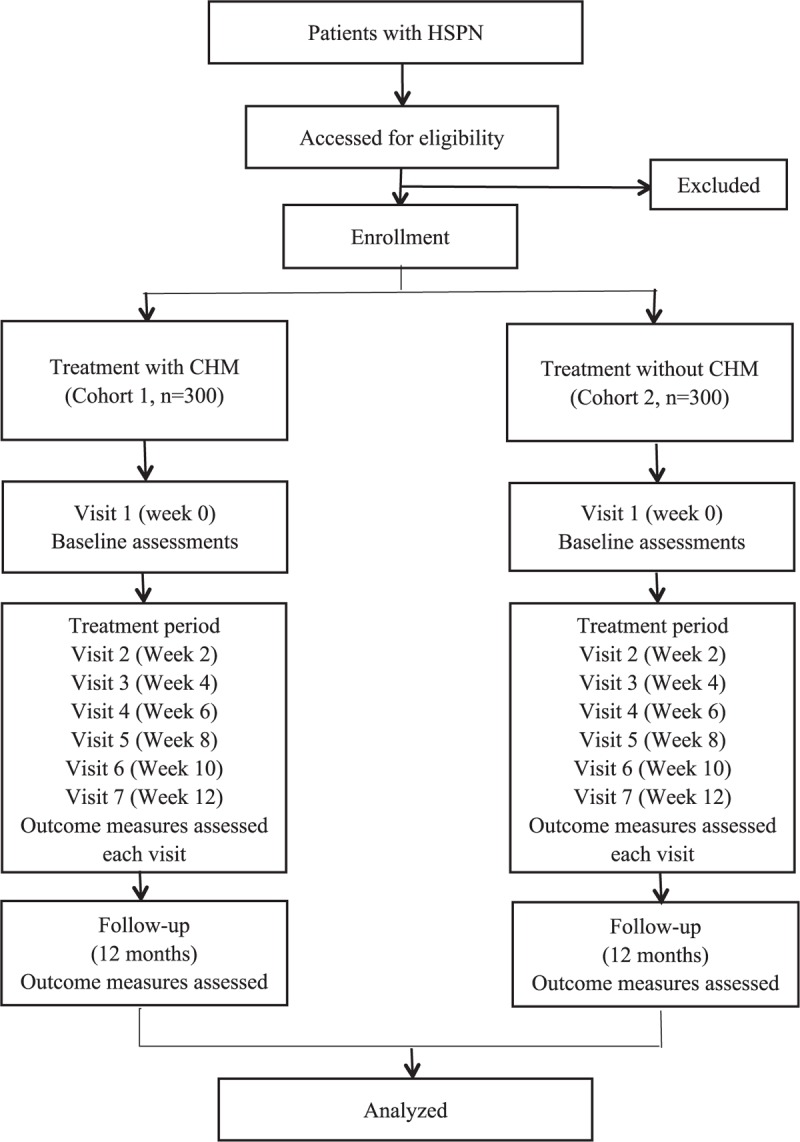
Protocol for selection participants. CHM = Chinese herbal medicine, HSPN = Henoch–Schönlein purpura nephritis.

HSPN patients are being recruited from outpatient and inpatient departments of three large comprehensive hospitals in China, including the Affiliated Hospital of Liaoning University of TCM, the First Affiliated Hospital of China Medical University, and the Shengjing Hospital of China Medical University. The patient screening will be conducted by clinicians who are not involved in the study. If a patient meets the study criteria, the clinician who is responsible for the patient screening will provide him/her with written information, explain the study in detail in an understandable language and obtain the written consent if he/she is willing to take part in the study. Informed consent must be obtained for all patients enrolled in the study. The eligible children with HSPN taking CHM in one of the above 3 hospitals were classified as cohort 1 and participants received WM without CHM belong to cohort 2. Trials that combine CHM with WM will be included, if the cohort 2 received the same WM as the cohort 1. Doctors of traditional Chinese medicine in the above hospitals prescribe CHM for patients according to their symptoms and signs, tongue manifestations, and pulse condition. The study flow chart is shown in Figure 1.

### 2.3 Ethical issues

This study registered in ClinicalTrials.gov with approval number NCT02878018 and was approved by the Ethics Committee of the Institutional Ethics Board of the Affiliated Hospital of Liaoning University of Traditional Chinese Medicine (Shenyang, China; Approval number: 2016CS(KT)-002-01). After completing a baseline description of the study to the enrolled subjects, written informed consent will be obtained from each of patients.

### 2.4 Participants

#### 2.4.1 Diagnosis criteria

Diagnosis and treatment guidelines for HSPN was based on the criteria in the 2000 Congress of Chinese Pediatric Society.^[21]^

#### 2.4.2 Inclusion criteria

1. A diagnosis of HSPN according to the diagnosis criteria.
2. Age: 5 to 18 years old (including 5 and 18 year old).
3. The clinical classification of HSPN includes isolated hematuria, insolated proteinuria, hematuria with proteinuria, and acute glomerulonephritis.
4. The ability to provide detailed connection and complete a follow-up.
5. The ability to understand and sign a written informed consent.

#### 2.4.3 Exclusion criteria

1. HSPN with renal insufficiency.
2. A clinical classification of HSPN that includes nephritic syndrome, rapidly progressive glomerulonephritis, and chronic glomerulonephritis.
3. Suffering from serious complications, such as respiratory, digestive, hematological, or liver diseases.
4. Tumor, infectious diseases, or mental disorders.
5. Allergic to TCM use.
6. No prescribed medication, poor compliance, or incomplete data affecting the efficacy and safety of these judgments.
7. A history of another clinical trial in the previous 2 weeks.
8. No consent form signed.

### 2.5 Outcome measures

#### 2.5.1 Primary outcomes

The primary outcomes of this study are remission rate and recurrence rate of HSPN.

#### 2.5.2 Secondary outcomes

1. Symptom improvement (such as urine erythrocyte, urine protein, etc)
2. Adverse events.

Each symptom or sign will be given a score, and the sum of all scores will be recorded in Table 1.

**Table 1:**
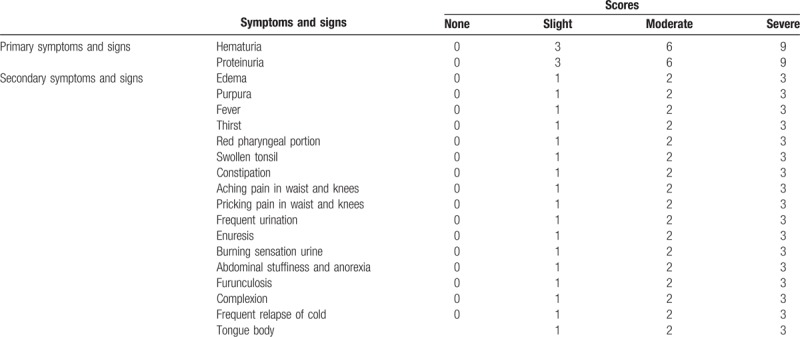
Scores for symptoms and signs.

### 2.6 Sample size calculation

According to the response rates of treatment, the cohort 1 and the cohort 2 were 53% and 40%, respectively. It was estimated that 250 patients of each group would be sufficient to achieve 90% power in detecting treatment differences, based on a 2-sided alpha at a significance level of 0.05. Meanwhile, considering a dropout rate of 20%, we concluded that a total of 300 patients for each cohort would need to be recruited to ensure significant results.

### 2.7 Safety assessment

For the sake of participant safety, the following biological indicators will be monitored and watched throughout the trial: blood routine, liver function (AST, ALT), renal function (BUN, Cr), and electrocardiography (ECG). Any unexpected symptoms and signs or feelings of discomfort in patients will be recorded as adverse events. For each adverse event, the starting date, ending date, degree, relationship to the trial and potential to trigger patient drop-out from the study will be carefully recorded and considered. If an adverse event occurs, the patient will be monitored until his/her adverse event disappears.

### 2.8 Data collection

Both cohorts undergo a 12-week treatment and 12-month follow-up. Case report forms (CRFs) contain a range of information, and these will be completed by the corresponding researchers at each center. The recruited patients will be asked to visit the clinicians 7 times for the assessment of the treatment efficacy during the treatment period (at baseline and in the 2nd, 4th, 6th, 8th, 10th, and 12th weeks after the first treatment). During the follow-up period, patients will be asked to visit once a month. If a patient fails to visit during one of the follow-up observations, the assessor will contact him/her by phone (Table 2). All data collected using paper-based CRFs will be submitted to an online clinical research data-capture computer system developed by the research group (http://59.46.50.9:9889). Only the members from the research team will have access to these data.

**Table 2:**
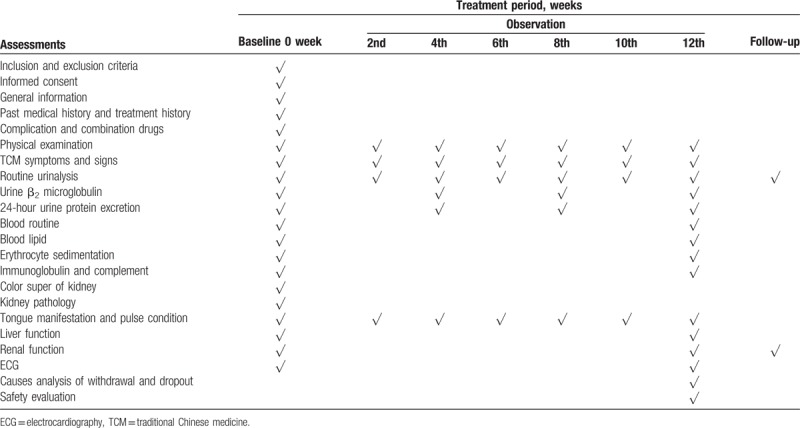
Calendar summary.

### 2.9 Statistical analysis

The statistical analysis will be conducted by a statistician of the Institute of Basic Research in Clinical Medicine, China Academy of Chinese Medical Sciences, Beijing, China. The statistician is blinded to the entire trial and will use SAS 9.1.3 (SAS Institute, Cary, NC, USA) and SPSS Version 13.0 (SPSS Inc., Chicago, IL) software. To test the hypotheses about associations between baseline risk factors and HSPN incidence, hazards ratios will be computed using the Cox proportional hazard regression models. Baseline differences between the groups will be assessed with the use of Student's *t*-test for normally distributed continuous variables and the nonparametric Mann–Whitney *U*-test for non-normally distributed variables. Comparisons between the groups will be conducted using an analysis of covariance (ANCOVA) with the baseline as the covariate. For efficacy analysis, changes from the baseline to the endpoint of treatment in scores will be tested with repeated measured analysis of variance (ANOVA). Within each group, differences will be assessed with paired t-test for normally distributed data and the Wilcoxon signed-rank test for non-normally distributed data. Safety will be assessed by participant compliance and adverse events. For analysis of the final dataset, missing data will be filled in using the last observation carried forward (LOCF) approach. A *P* value <.05 is considered significant.

## 3 Discussion

HSPN is a major public health problem and accounts for 78.9% of secondary glomerulopathy in children.^[22]^ Currently, TCM plays a considerable role in symptom and quality of life improvement for HSPN children patients in China and worldwide. In this study, we propose the use of CHM to improve the clinical management of HSPN. A prospective cohort design is employed in the study, and 600 HSPN children patients will be treated as subjects.

To guarantee the quality of the study programs, only patients who meet the inclusion criteria will be recruited. In addition, patients will be excluded if they also suffer from nephritic syndrome, rapidly progressive glomerulonephritis, chronic glomerulonephritis, respiratory diseases, digestive diseases, and liver or mental diseases. The medical therapies associated with these afflictions may interact with the planned interventions, resulting in difficulty in interpreting the clinical results.

It is worth mentioning that we adopted an online clinical research data-capture computer system to collect the data. To minimize the bias and artificial errors of human data management, each patient record will be typed by three researchers and verified before storing. Once stored, the data will be visible to the statisticians and supervisors of this study. They will not be able to modify the data entries. The clinical appraisers and statisticians will be kept unaware of whether a participant belongs to the experimental or the control group. We attempt to establish the chronic disease management programs incorporating information technology (IT) solutions for children with HSPN and explore the effective model for long-term treatment through disease control, psychological intervention, and social support.

The outcomes in the follow-up will include renal events, mortality rate, and adverse events. These outcomes will be easy to assess with only a few questions. The physicians who follow the entire process of the study are familiar with patients and are experienced interviewers. These measures will guarantee the quality of the follow-up. In conclusion, this prospective cohort study will identify whether CHM is helpful for the treatment of HSPN in pediatric patients. The results of this research may generate scientific evidence for further confirmatory studies.

In this trial, we intend to assess the efficacy and advantage of the use of CHM for the treatment of children with HSPN. We hypothesize that CHM will be an effective and safe way to treat HSPN patients and to reduce the recurrence rate.

## Acknowledgments

We thank Basic Research in Clinical Medicine, China Academy of Chinese Medical Sciences, Beijing, China, for their cooperation and technological support of statistical analysis. We appreciate the help and efforts for all research children participating in this trial. We also acknowledge Affiliated Hospital of Liaoning University of TCM, First Affiliated Hospital of China Medical University, and Sheng-Jing Hospital of China Medical University for their helpful support in treatment facilities, recruiting and treating patients. This trial was supported financially by the 2015 National Scientific Research Specific of TCM Industry (Number 201507001-03).

## Author contributions

JZ and SP wrote the first draft of the article of the study protocol. JL, XB, and FY are responsible for writing-review and editing YW, HJ, JZ, and SZ are responsible for managing for the project and conducting formal analysis. SP and GY are responsible for data curation. JZ received the funding to ensure this study is underway. All authors have contributed to the design and implementation of the study.

**Data curation:** Shuang Pang, Guanqi Yang.

**Formal analysis:** Jun Zhang, Yubin Wu, Hong Jiang, Shaoqing Zhang.

**Funding acquisition:** Jun Zhang.

**Project administration:** Jun Zhang, Yubin Wu, Hong Jiang, Shaoqing Zhang.

**Writing – original draft:** Jun Zhang, Shuang Pang.

**Writing – review & editing:** Jing Lv, Xiaohong Bai, Fang Yuan.
